# A Review of Deep Learning Methods for Multimodal Medical Image Fusion

**DOI:** 10.3390/s26144632

**Published:** 2026-07-22

**Authors:** Yanan Shi, Maria Rigou

**Affiliations:** Department of Management Science and Technology, University of Patras, 26334 Patras, Greece; yananshi857@gmail.com

**Keywords:** deep learning, medical image fusion, multimodal fusion, review

## Abstract

Multimodal medical image fusion (MMIF) aims to integrate complementary information from different imaging modalities into a single, more informative image to support clinical diagnosis. Since 2017, a growing number of deep learning-based approaches have been proposed for MMIF, including various network architectures designed to enhance visual quality. However, a comprehensive and up-to-date review of deep learning-based MMIF techniques is still lacking. To fill this gap, this paper provides a comprehensive survey of deep learning-based MMIF methods. First, we categorize MMIF approaches based on deep learning frameworks and conduct an in-depth analysis of loss functions, evaluation metrics, medical imaging modality pairs, and medical datasets. Then, we review the mainstream deep learning-based MMIF methods, including CNNs, Autoencoders, GANs, and Transformers. Subsequently, we introduce emerging deep learning methods, including diffusion models and Mamba-based methods. In addition, we summarize widely used datasets and evaluation metrics, conduct quantitative experiments on representative methods, and propose a unified set of evaluation metrics for standardized comparison. Finally, we identify key research challenges and outline promising future directions for deep learning-based MMIF. This survey aims to provide researchers with a clear understanding of recent progress in deep learning-based MMIF and to facilitate further studies in this area.

## 1. Introduction

Multimodal medical image fusion (MMIF) has received significant attention in recent years. Owing to the complementary properties of different medical imaging sensors, MMIF has been widely used in disease diagnosis [1,2], surgical navigation [3], and treatment planning [4]. Specifically, computed tomography (CT) images clearly reflect anatomical structures but offer limited soft tissue contrast. Magnetic resonance imaging (MRI) images provide high-resolution soft tissue contrast but are less effective for visualizing calcified tissues. Positron emission tomography (PET) and single-photon emission computed tomography (SPECT) images provide metabolic and functional information, but their low spatial resolution limits anatomical detail. MMIF aims to fuse complementary information from different imaging modalities into a single image with more comprehensive information, thereby improving diagnostic efficiency and accuracy.

Before the emergence of deep learning-based methods, various traditional methods had been proposed for MMIF. Traditional MMIF methods can generally be divided into three categories, including multiscale transform-based approaches [5,6,7,8], sparse representation techniques [9,10,11], and hybrid methods [12,13]. Although traditional image fusion methods exhibit stable fusion performance, activity level measurements and fusion rules need to be designed in a handcrafted manner, which limits their adaptability to complex fusion scenarios.

**Figure 1:**
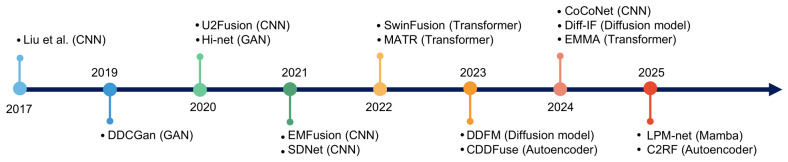
Milestones in deep learning-based MMIF methods from 2017 to 2025. Parentheses indicate the architecture each method is based on. CNN-based methods: Liu et al. [14], U2Fusion [15], EMFusion [4], SDNet [16], and CoCoNet [17]. GAN-based methods: DDcGAN [18] and Hi-net [19]. Transformer-based methods: SwinFusion [20], MATR [21], and EMMA [22]. AE-based methods: CDDFuse [23] and C2RF [24]. Diffusion model-based methods: DDFM [25] and Diff-IF [26]. Mamba-based method: LPM-net [27].

In recent years, deep learning has demonstrated remarkable performance in MMIF due to its superior feature representation ability. Liu et al. [14] were the first to use deep learning in MMIF, introducing a siamese convolutional neural network (CNN) to learn a mapping from source images to a weight map. Since then, research on deep learning-based MMIF methods has grown rapidly. Figure 1 presents a timeline of deep learning-based MMlF approaches, highlighting major milestones in the field. These approaches can be classified into CNN-based, GAN-based, Autoencoder-based, Transformer-based, diffusion model-based, and Mamba-based methods.

However, the current literature lacks a comprehensive review focusing on deep learning-based MMIF, which limits researchers’ understanding of recent advances and emerging trends, particularly for those new to the field. Several review papers have been published in the field of MMIF. Hermessi et al. [28], Azam et al. [29], and Ullah Khan et al. [30] covered both traditional and deep learning-based MMIF methods, but provided only limited coverage of the latter. Zhou et al. [31] categorized deep learning-based MMIF methods, but their review did not cover studies published from 2022 to 2025.

To address the limitations of existing surveys, this review provides a comprehensive overview of deep learning-based multimodal medical image fusion methods published from 2017 to 2025. The main contributions are summarized as follows:

1. We establish a taxonomy covering CNNs, Autoencoders, GANs, Transformers, diffusion models, and Mamba models. Based on this taxonomy, we analyze 49 representative methods in terms of architectural designs, loss functions, evaluation metrics, modality combinations, and datasets.
2. We recommend a unified evaluation metric set comprising VIF, MI, SSIM, SCD, and SD. These metrics assess visual fidelity, information preservation, structural similarity, correlation between source and fused images, and image feature characteristics, respectively. We further compare nine representative methods from five architectural categories using the same dataset and evaluation protocol.
3. We identify the major limitations of current MMIF research and discuss future directions, including improved evaluation metrics, registration-aware fusion, Mamba-based methods, benchmark expansion, application-driven fusion, generalizable MMIF models, and clinical translation.

**Figure 2:**
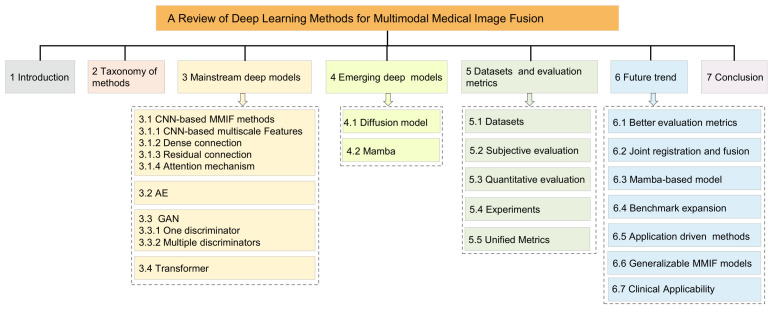
Overall framework of the review.

The overall framework of this paper is illustrated in Figure 2. Section 2 introduces the taxonomy of deep learning-based MMIF methods. Section 3 and Section 4 review mainstream and emerging approaches, respectively, while Section 5 summarizes datasets and evaluation metrics, presents quantitative experiments on representative methods, and proposes a unified set of evaluation metrics. Section 6 discusses future research directions, and Section 7 concludes the paper.

## 2. Deep Learning-Based MMIF Method Classification and Fusion Metric Analysis

A typical deep learning-based MMIF pipeline consists of three stages: feature extraction, feature fusion, and image reconstruction. In the first stage, features are extracted from the medical images. These features are then fused in the second stage. Finally, the fused image is reconstructed in the final stage.

In this review, deep learning-based MMIF methods are categorized into two major groups: mainstream and emerging methods. The mainstream approaches are further classified into four subcategories according to their backbone architectures: CNN-based, GAN-based, Autoencoder-based, and Transformer-based methods. The emerging deep learning-based MMIF methods include diffusion model-based and Mamba-based methods. A summary of representative MMIF methods is presented in Table 1.

We present the key trends identified from the review of 49 selected papers in the field of MMIF. Our goal is to provide a comprehensive overview of the current research landscape. We analyze the frequencies of loss functions, evaluation metrics, modality pairs, and datasets used in these studies, with each frequency calculated as the number of papers in which each item appears divided by the total number of papers (i.e., 49).

**Figure 3:**
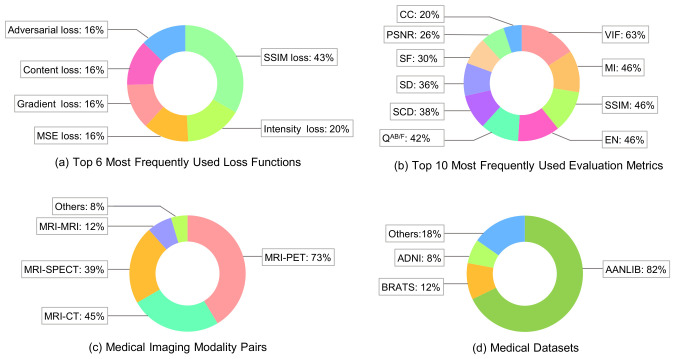
Distribution of loss functions, evaluation metrics, imaging modality pairs, and datasets in the reviewed papers listed in Table 1. The percentages denote the frequency of use of each item among these papers.

Loss functions: In deep learning-based image fusion, the choice of loss function is critical, as it directly influences the optimization of fusion performance. The six most commonly used loss functions are SSIM, intensity, MSE, gradient, content, and adversarial losses, as shown in Figure 3a.

Evaluation metrics: Choosing appropriate objective evaluation metrics is essential for the assessment of medical image fusion, supporting practical deployment and promoting scientific progress in the field. There is no standardized evaluation metric for MMIF, which leads to the use of different metrics across studies. Figure 3b shows the top ten most frequently used evaluation metrics, which are VIF, MI, SSIM, EN, $Q^{\mathrm{AB}/F}$, SCD, SD, SF, PSNR, and CC.

Medical imaging modality pairs: The MRI-PET modality pair is dominant in imaging techniques, with 73% of the 49 papers utilizing this combination. The next most common pairs are MRI-CT, followed by MRI-SPECT and MRI-MRI. The “Other” category includes less frequent modality pairs, such as CT-PET and GFP-PC, as shown in Figure 3c.

Medical datasets: The most commonly used datasets are AANLIB (82%), BRATS (12%), and ADNI (8%). The “Other” category also includes less frequently used datasets, such as Brain Atlas, ISLES, and ECSSD, as illustrated in Figure 3d.

## 3. Mainstream Deep Learning-Based MMIF Methods

### 3.1. CNN-Based MMIF Methods

CNNs have emerged as one of the most widely adopted architectures for improving performance in MMIF. Liu et al. [14] first introduced a CNN-based MMIF method. Since then, a wide variety of CNN-based methods have emerged. These methods can generally be categorized into four groups: multiscale feature mechanisms, dense connection-based approaches, residual connection-based methods, and attention mechanisms.

#### 3.1.1. CNN-Based Multiscale Feature Mechanisms

CNN-based multiscale feature mechanisms are widely applied in MMIF. These mechanisms employ convolutional operations with different receptive field sizes or spatial downsampling to extract features at multiple scales. Based on the implementation strategy, multiscale mechanisms for MMIF can be grouped into three types: downsampling-based mechanisms, convolutional kernel-based mechanisms, and hybrid mechanisms that combine both downsampling and multi-kernel convolutions.

**Figure 4:**
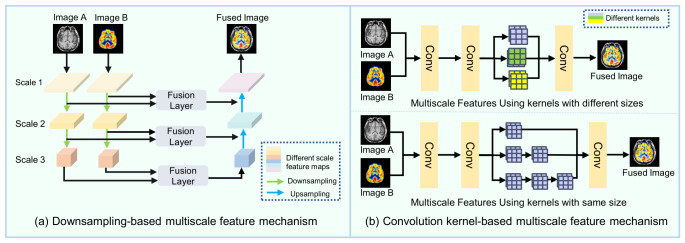
Convolution kernel-based multiscale feature mechanism for MMIF.

Downsampling-based multiscale feature mechanisms: In MMIF methods, downsampling-based multiscale mechanisms typically involve the following steps: First, the source images are fed into a multi-branch feature extractor, where each branch performs downsampling through pooling layers or strided convolutions (with stride > 1) to extract features at multiple scales. Subsequently, the features at the same scale are fused. Finally, the fused features are transmitted to the corresponding scales in the image reconstruction layer via skip connections, where upsampling operations are performed to reconstruct the final image, as shown in Figure 4. The downsampling-based multiscale mechanism utilizes both low-level fine details from shallow networks and high-level semantic features from deep networks, effectively enhancing the fusion quality [66].

Several representative MMIF methods have adopted this downsampling-based multiscale strategy [17,32,33]. For instance, CoCoNet [17] proposes a contrastive learning network that incorporates multiscale features. Its feature extractor consists of three branches: one leverages max pooling to obtain multiscale features, while the other two employ VGG19 [67] as the backbone to extract features at different scales. MM-net [32] adopts a dual-branch multiscale feature extraction strategy based on MixFormer [68], in which each branch consists of five stages with different downsampling rates to extract local and global features across scales. Similarly, MRSCFusion [33] uses max-pooling-based downsampling to extract multiscale deep features from stacked convolutional blocks.

Convolution kernel-based multiscale feature mechanism: An effective method for obtaining multiscale features is to employ convolution kernels of varying sizes, as illustrated in Figure 4. For example, Tang et al. [24] present C2RF, an AE-based framework for multimodal image registration and fusion that employs three multiscale residual blocks to extract shallow features in the feature encoder. The multiscale residual block contains three branches, with convolution kernels of sizes 5×5, 3×3, and 1×1. Similarly, Guo et al. [34] propose SMAFusion, which employs a multiscale feature encoder to capture both local and global features across different scales.

Another way of achieving multiscale features is to use same-size convolution kernels, because stacking two 3×3 convolutions approximates the receptive field of a 5×5 kernel, while stacking three approximates that of a 7×7 kernel [36], as illustrated in Figure 4. These approaches can achieve comparable performance with fewer parameters, making the network more efficient. For example, MATR [21] introduces a three-branch multiscale feature extractor based on adaptive convolutions and transformers. The first branch employs a single 3×3 adaptive convolution, while the second and third branches stack two and three such modules, respectively, to approximate the receptive fields of 5×5 and 7×7 kernels. Recently, FDGNet [35] introduces a feature difference guided network that consists of two parallel branches of stacked 3×3 convolutions, in which feature differences are computed at each scale and transmitted to deeper layers through skip connections.

**Figure 5:**
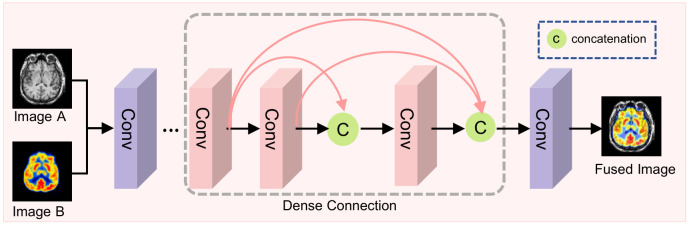
Image Fusion Methods Based on Dense Connection. All arrows indicate the direction of information flow.

Hybrid multiscale features mechanism: Some methods combine downsampling with convolutional kernels to effectively capture multiscale features [36,37,38], as shown in Figure 5. For instance, Fu et al. [36] propose a multiscale residual pyramid attention network, which performs downsampling at multiple spatial resolutions and applies parallel branches of stacked 3×3 convolutions to approximate 5×5 and 7×7 receptive fields. MdAFuse [37] captures multiscale representations by combining downsampling operations with convolutional layers of varying kernel sizes, including 1×1, 3×3, 5×5, and 7×7.

#### 3.1.2. Dense Connection-Based MMIF Methods

Dense connection-based MMIF methods refer to architectures in which feature maps from each convolutional layer are passed to subsequent layers. Each layer receives concatenated inputs from preceding layers, as illustrated in Figure 5. By fully exploiting intermediate-layer features, such methods enhance feature propagation, promote feature reuse, and mitigate gradient vanishing [4,69].

Owing to these advantages, many MMIF methods based on dense connections have been proposed. For instance, Xu et al. [15] develop U2Fusion, which employs a DenseNet module to generate the fused image. The architecture of the DenseNet comprises multiple densely connected blocks to improve fusion performance. Zhang et al. [16] propose SDNet, which consists of a squeeze network and a decomposition network. Dense connections within the squeeze network are employed to reduce information loss. EMFusion [4] applies dense connections in the FusionNet module to create short connections among the first four convolutional layers. MUFusion [39] uses two densely connected feature extraction blocks to capture deep features from the input images.

#### 3.1.3. Residual Connection-Based MMIF Methods

**Figure 6:**
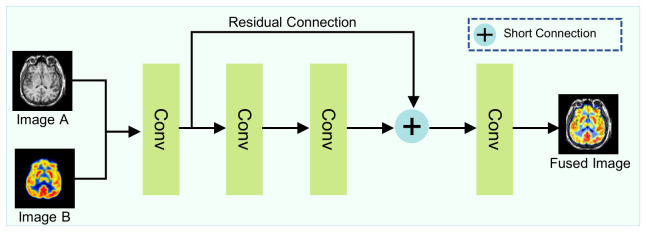
Image Fusion Methods Based on Residual Connection.

Residual connections are highly effective in deep learning-based MMIF methods. They utilize skip connections to directly add the input features to the output of subsequent convolutional layers as the final feature output, as illustrated in Figure 6. Residual connections in image fusion facilitate the utilization of multi-level information, enhance feature representation, and mitigate gradient vanishing.

Residual connections can be employed in different stages of the fusion process. For example, Liu et al. [41] employ a residual block to process the fused features in the image reconstruction stage. Liu et al. [40] propose an adaptive fusion strategy based on adaptive integration weight maps, utilizing residual blocks to construct the base weight map integration. Fu et al. [36] introduce a multiscale residual pyramid attention module for feature extraction, which uses a residual attention mechanism to enhance training efficiency and enhance feature representation quality.

#### 3.1.4. Attention Mechanism-Based MMIF Methods

**Figure 7:**
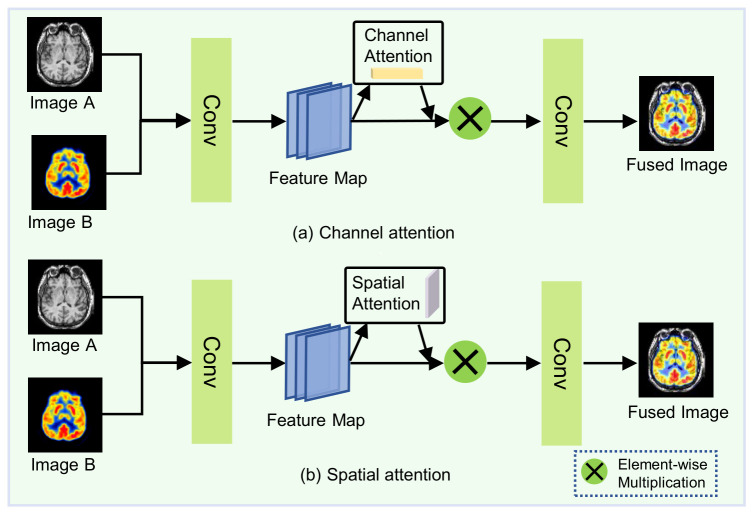
Attention Mechanisms in CNN-based MMIF.

Attention mechanisms have been widely used in CNN-based MMIF methods. The principle of attention mechanisms is to dynamically assign varying weights to different elements of the feature maps, enabling the network to focus on the most informative elements and thus improve performance in MMIF. Attention mechanisms in CNN-based MMIF are generally categorized into spatial attention [41,42,43] and channel attention [23,44,45], as shown in Figure 7.

Attention mechanisms can be employed at different stages of MMIF approaches. In some methods, attention mechanisms are used for feature extraction. For instance, MSAIF-net [42] introduces a multistage spatial attention mechanism that captures long-range contextual information from each image and adaptively assigns attention weights at different stages within the feature extraction module. In other approaches, attention mechanisms are used for feature fusion. For instance, Liu et al. [41] propose an attention-based feature fusion module to enhance feature learning, where parallel spatial and channel attention branches jointly contribute to the fusion process. Di et al. [45] also propose an AE-based MMIF method that incorporates spatial and channel attention mechanisms to guide the feature fusion process more effectively.

CNN-based models exhibit strong local feature extraction capability due to inherent inductive biases such as locality and translational invariance. In terms of computational complexity, convolution operations are generally efficient and highly parallelizable, and their computational cost increases mainly with image resolution, kernel size, and channel number, making CNN-based models relatively practical for image fusion tasks. Nevertheless, the limited receptive field of convolution operations restricts long-range dependency modeling and global context aggregation, which may reduce their effectiveness in capturing complex structural relationships for image fusion.

### 3.2. AE-Based MMIF Methods

**Figure 8:**
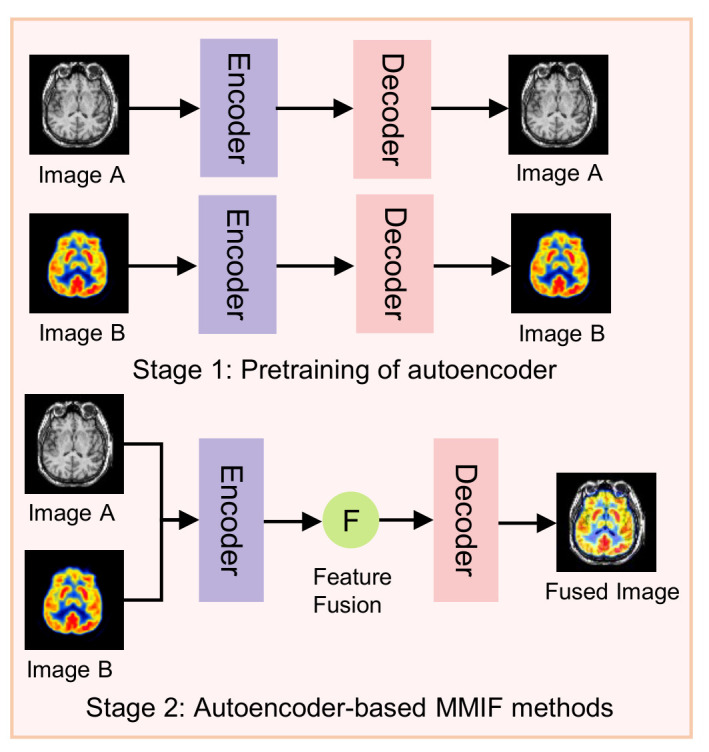
Image Fusion Methods Based on Autoencoder.

AE-based MMIF methods typically involve two stages. In the first stage, an autoencoder is trained on a medical image dataset such as the Harvard Whole Brain Atlas or on a natural image dataset such as the Microsoft Common Objects in Context (MS COCO). In the second stage, the encoder and decoder previously trained are employed for feature extraction and image reconstruction, respectively. The feature fusion between the encoder and decoder is achieved either through hand-crafted fusion strategies or a secondary training step. An overview of these stages is illustrated in Figure 8.

IFSepR [46] proposes a general three-stream AE-based method to disentangle common and private features. A feature-space adaptive fusion rule is utilized for feature fusion to preserve complementary information. UNIFusion [47] introduces a fusion strategy that integrates features using averaging, maximum selection, spatial attention, and gradient perception. SGFusion [48] utilizes an addition strategy to integrate the features extracted during the encoding of the source images.

However, the above-mentioned methods adopt handcrafted fusion strategies, which may be limited in performance. To overcome these limitations, some methods propose an additional learning stage specifically designed for feature fusion [23,24]. For example, CDDFuse [23] designs a fusion layer that combines transformer-based global components with invertible neural network (INN)-based local components. In C2RF [24], the extracted common features are fused through convolutional feature concatenation, while the unique features are integrated using a pixel-wise attention mechanism.

Although AE-based MMIF methods have shown promising results, their performance is often constrained by the limited availability of medical image datasets. Some studies address this issue by pretraining autoencoders on large-scale natural image datasets and then fine-tuning them on medical datasets. Nevertheless, the structural and textural differences between natural and medical images may weaken cross-domain transferability. Therefore, constructing large-scale, well-aligned multimodal medical image datasets is essential for improving the generalization and reliability of AE-based MMIF methods.

### 3.3. GAN-Based MMIF Methods

**Figure 9:**
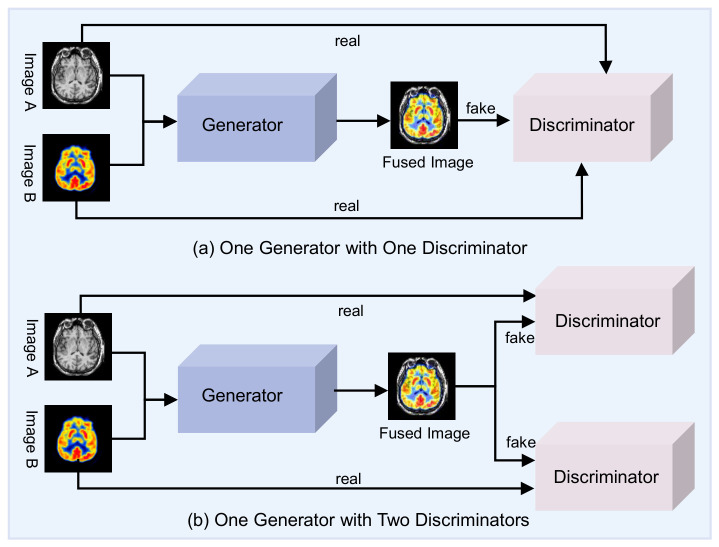
Architecture of GAN-Based Image Fusion Methods.

GANs have been widely adopted in computer vision tasks since their introduction in 2014 [70]. GAN-based MMIF methods aim to learn the data distribution of medical images through adversarial learning. Typically, a GAN-based MMIF method consists of two modules: the generator aims to produce fused images to deceive the discriminator, while the discriminator is responsible for distinguishing real from generated data. Both modules are continuously optimized through adversarial training, enabling the model to progressively improve the quality of the fused medical images. Existing GAN-based MMIF methods can be divided into two categories: one generator and one discriminator, and one generator and multiple discriminators, as illustrated in Figure 9.

#### 3.3.1. One Generator and One Discriminator

Zhou et al. [19] propose Hi-Net, which consists of three components: a modality-specific network, a fusion network, and a synthesis network. The synthesis network contains a generator responsible for generating fused outputs and a discriminator that distinguishes generated images from real images. BMGAN [49] introduces a 3D end-to-end network, where the generator incorporates a bi-directional mapping mechanism and a 3D Dense-UNet to enhance the visual fidelity of synthetic images. This design effectively mitigates the limitations of conventional GANs in encoding meaningful latent representations and maintaining structural integrity in the fused output. MGM-GAN [50] constructs its generator based on multiscale convolutional modules and a gating mechanism to integrate more information into the fused image. Recently, PRRGAN [51] introduces a tri-modal generative adversarial network for MMIF, in which the generator is composed of multiscale residual modules and inference attention blocks.

#### 3.3.2. One Generator and Multiple Discriminators

A major limitation of one-discriminator GANs is their tendency to generate fused images biased toward one modality, which may cause the loss of complementary information from the other modality and lead to modality imbalance. To address this problem, some researchers have proposed GANs based on two discriminators [1,18,52,53]. Ma et al. [18] introduce DDcGAN, which adopts two discriminators to discriminate the structural differences between the fused image and the source images, and integrates a content loss to guide optimization. Zhou et al. [52] propose a generator with an encoder-decoder architecture and a dual-discriminator to evaluate gradient and intensity consistency in the fused image. Gradient-based and intensity-based loss functions are also introduced to further guide the training process. TCGAN [53] embeds a wavelet transform-based pre-fusion module into the GAN framework to introduce domain-specific priors. The model employs a multiscale CNN–Transformer hybrid as the generator and utilizes dual Transformer-based discriminators to capture complementary local and global features. Departing from conventional approaches that adopt classification networks as discriminators, Liu et al. [1] employ a semantic segmentation network to distinguish between fused and source images, enabling more fine-grained evaluation.

In addition to the aforementioned dual-discriminator approaches, MHW-GAN [54] adopts a GAN architecture comprising one generator and three discriminators for general image fusion tasks. Besides the two discriminators corresponding to the two source images, a third discriminator is introduced to evaluate the consistency of the fused edge image with the joint edge representation.

GANs with multiple discriminators can better preserve the distinctive features of each source image and improve fusion performance. However, the use of multiple discriminators increases the training cost by introducing additional parameters, adversarial losses, and optimization steps, although the inference cost mainly depends on the generator. In addition, training such models remains challenging due to instability and mode collapse.

### 3.4. Transformer-Based MMIF Methods

**Figure 10:**
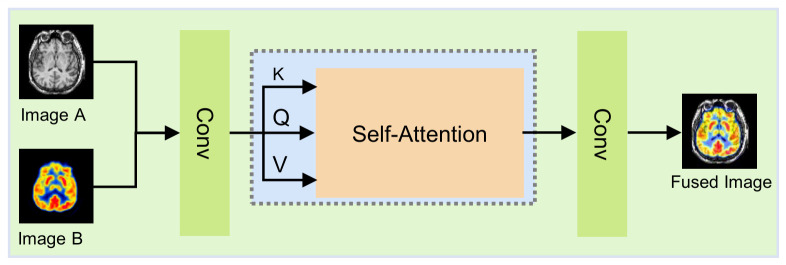
Architecture of Transformer-based image fusion methods. K, Q, and V denote Key, Query, and Value, respectively.

Transformers leverage the self-attention mechanism to effectively capture long-range dependencies and global semantic information, demonstrating remarkable success in natural language processing and computer vision tasks. Transformer models were first introduced to the field of image fusion in 2021. Since then, many Transformer-based MMIF methods have been proposed. Transformers are usually integrated with CNNs in image fusion to leverage their complementary advantages: CNNs are effective in extracting local spatial features, while transformers specialize in modeling long-range dependencies. An overview of a typical hybrid CNN-Transformer architecture is illustrated in Figure 10. Transformers are often applied to different stages of MMIF methods, including feature extraction, fusion, and reconstruction.

Transformers for feature extraction: In some MMIF methods, Transformers are applied to feature extraction [21,23,32,59]. For example, CDDFuse [23] proposes a dual-branch Transformer-CNN architecture in the feature extraction module. Specifically, both Restormer [71] and Lite transformer [72] are used to extract shared features and private features. MATR [21] integrates adaptive convolutions and adaptive transformers to enhance semantic extraction capability. MM-Net [32] proposes a multiscale fusion method based on MixFormer [68], which effectively captures both local features and global contextual information across multiple scales, thereby improving feature representation quality.

Transformers for feature fusion: In addition to feature extraction, Transformers have also been employed in the fusion stage of MMIF frameworks to effectively integrate information from different modalities [33,55,56,57]. For example, MRSCFusion [33] employs a dual-branch fusion strategy combining Transformer and CNN modules to integrate multiscale features. The global branch incorporates Swin Transformer blocks and residual connections to model high-level contextual dependencies, while the local branch utilizes densely connected convolutional layers to preserve fine-grained spatial details. FATFusion [56] employs a Transformer-based guidance module to promote effective feature transmission and aggregation, thereby improving the integration of global contextual information.

Transformers across multiple stages: Transformers have also been applied to multiple stages of MMIF methods [20,22,58]. For instance, EMMA [22] employs four stacked Transformer–CNN blocks across both the feature extraction and image reconstruction stages. SwinFusion [20] is a representative full-stage fusion model built upon the Swin Transformer architecture. It integrates self-attention and cross-attention to capture intra-modal and inter-modal dependencies. It represents one of the earliest studies to explicitly visualize global information flow in image fusion.

While most transformer-based MMIF methods adopt hybrid CNN–Transformer architectures, a few recent studies have begun exploring the integration of transformers with MLPs [59] or diffusion models [60]. These efforts suggest a growing interest in developing alternative architectures beyond CNNs. Despite their advantages in capturing long-range dependencies and extracting global contextual information, Transformer-based methods usually introduce high computational complexity and memory consumption due to the attention mechanism, which presents challenges for deployment in environments with limited resources.

## 4. Emerging Deep Learning-Based MMIF Methods

### 4.1. Diffusion Model-Based MMIF Methods

The diffusion model, first proposed in 2014 as a family of generative models, has recently surpassed GAN-based approaches in tasks such as image generation [73] and image restoration [74], thereby challenging the long-standing dominance of GANs in this field. DDFM [25] was the first method to introduce diffusion models into MMIF in 2023. Since then, an increasing number of diffusion model-based MMIF methods have been proposed, showing promising performance.

To illustrate the progress in this domain, we briefly review three notable diffusion model-based MMIF frameworks. UUD-Fusion [62] introduces a two-stage general image fusion method based on diffusion model. In the first stage, the CNN generates a rough fusion result. In the second stage, two diffusion-based sampling algorithms are employed to explore the solution space and refine the fusion quality. Diff-IF [26] leverages the distribution information provided by the fusion knowledge prior and targeted search to construct the forward diffusion process, while the reverse diffusion process generates fused images that conform to the fusion knowledge prior distribution. FusionDiff [61] addresses the data dependency issue in existing unified image fusion approaches by pre-fusing source images through a diffusion process. It introduces a noise prediction network, a Spatially-Adaptive Constraint layer, and a skip-sampling mechanism to improve image quality while reducing the computational cost of traditional diffusion models.

Overall, diffusion models offer notable advantages, including high-quality generation, stable training, and a mathematically interpretable modeling process. However, both training and inference usually require substantial computational resources and time.

### 4.2. Mamba-Based MMIF Methods

Mamba [75], a recently introduced selective state space model (SSM), has received increasing attention for its ability to capture long-range dependencies with linear computational complexity.

Given these advantages, many Mamba-based MMIF methods have been proposed [27,63,64,65]. For example, LPM-Net [27] employs three processing branches, each integrating a dense CNN with Mamba to enhance feature extraction. Zhang et al. [64] propose a VSSM-based cross-domain image fusion framework with a dual-branch extractor and a VMamba-based decoder for image fusion and generation. DWMFusion [65] is designed as a lightweight MMIF framework that integrates discrete wavelet transform and convolution for feature extraction, and adopts a Mamba-based multilevel module to efficiently fuse multiscale complementary features.

## 5. Datasets and Evaluation Metrics

### 5.1. Datasets for MMIF

This section reviews the most commonly used public medical datasets, as shown in Figure 3d. These datasets encompass various imaging modalities, such as CT, MRI, and PET. They provide high-quality samples that facilitate the development of image fusion algorithms. The datasets are summarized in Table 2.

The Whole Brain Atlas (AANLIB), developed by Harvard Medical School, includes approximately 13,000 brain images from both healthy subjects and patients with neurological disorders such as cerebrovascular disease, brain tumors, and Alzheimer’s disease. It includes four imaging modalities: MRI, CT, PET, and SPECT, with all images offered in GIF format.

The Brain Tumor Segmentation (BRATS) dataset, part of the MICCAI BRATS Challenge series, consists of multimodal MRI scans from glioma patients, including four clinically relevant sequences: T1-weighted, T1 with contrast enhancement (T1Gd), T2-weighted, and FLAIR. The data are provided in the standardized NIFTI format. As of BRATS 2021, the dataset includes over 1250 cases, covering both low-grade and high-grade gliomas.

The Alzheimer’s Disease Neuroimaging Initiative (ADNI) dataset was developed to support research into the early diagnosis and progression of Alzheimer’s disease. It comprises multimodal imaging data from patients with Alzheimer’s disease, individuals with mild cognitive impairment, and cognitively normal controls. The dataset includes three imaging modalities: MRI, PET, and CT, which are used to capture various aspects of brain structure and function.

The Open Access Series of Imaging Studies (OASIS) is a publicly available brain imaging dataset developed by Washington University, focusing on Alzheimer’s disease and its prodromal stage, mild cognitive impairment. The dataset consists of four versions: OASIS-1, OASIS-2, OASIS-3, and OASIS-4. Among these, OASIS-3, which was released in 2019, represents the most comprehensive version and includes longitudinal structural MRI and PET scans acquired from more than 1000 participants.

The Cancer Imaging Archive (TCIA) is a publicly accessible medical imaging repository maintained by the U.S. National Cancer Institute (NCI). TCIA focuses on the collection and dissemination of multimodal cancer-related imaging data. TCIA offers datasets covering various cancer types from different anatomical sites, such as gliomas, lung cancer, breast cancer, liver cancer, and prostate cancer. The imaging modalities available include CT, MRI, PET, and ultrasound. All data are stored in the standardized DICOM format for consistency and interoperability.

### 5.2. Subjective Evaluation Criteria

Subjective qualitative evaluation assesses the quality of fused images through human visual perception, considering factors such as salient features, sharpness, and spatial detail. It captures the perceived visual quality and provides intuitive and reliable assessments. Consequently, it has been widely used in the evaluation of MMIF methods. However, subjective evaluation relies on individual judgment, and differences in evaluation criteria among observers may compromise the consistency [76] and reproducibility of the results [77]. To address these challenges, a variety of objective metrics have been proposed by researchers. These metrics enable quantitative and automated assessments of fusion quality, improving both the efficiency and reliability of the evaluation process.

### 5.3. Quantitative Evaluation Metrics

Objective quantitative evaluation does not rely on subjective human perception but assesses image quality through mathematical computation. It is efficient, consistent, and repeatable, and is less affected by visual bias. This section introduces several representative evaluation metrics commonly used in the surveyed papers and their corresponding categories. These metrics are listed in Table 3. We use A and B to denote the two source images and F to denote the fused image. X represents either source image A or B.

### 5.4. Experiments

To quantitatively compare representative deep learning-based MMIF methods, this study selects nine methods from the 49 reviewed methods, covering five major architectural categories: CNN-based methods [4,15], AE-based methods [23,24], a GAN-based method [18], Transformer-based methods [20,22], and diffusion model-based methods [25,26]. Experiments are conducted on 40 randomly selected PET–MRI image pairs from the public Harvard Whole Brain Atlas dataset. The 10 fusion metrics introduced in Section 5.3 are used to evaluate fusion performance. For all selected deep learning-based methods, the pretrained models released by the original authors are used, and no additional retraining is performed in this study.

Table 4 presents the quantitative results of the nine selected deep learning-based MMIF methods. The results show that different methods exhibit advantages under different metrics. Diff-IF [26] achieves the best VIF and SSIM, indicating better visual information preservation and structural consistency. Diff-IF [26] and DDFM [25] obtain the highest MI, suggesting effective preservation of complementary source information. DDFM [25] achieves the best PSNR and CC, reflecting lower reconstruction distortion and stronger source-image correlation. CDDFuse [23] obtains the best SD, SCD, and SF, indicating advantages in contrast representation, complementary information preservation, and spatial detail representation. EMMA [22] achieves the highest EN, while SwinFusion [20] obtains the best $Q^{\mathrm{AB}/F}$, reflecting their respective strengths in information richness and edge-related feature preservation. Overall, no single method consistently outperforms the others across all metrics, indicating the necessity of multi-metric evaluation combined with qualitative analysis.

### 5.5. Unified Evaluation Metrics

Numerous evaluation metrics have been proposed for image fusion. Based on [87,88], these metrics can be broadly classified into five categories: human visual quality metrics, information-based metrics, structural preservation metrics, image feature-based metrics, and correlation-based metrics. Despite the large number of proposed metrics, no single metric can comprehensively characterize all aspects of fusion performance. As shown in Table 1, existing studies typically employ only a subset of evaluation metrics, which may limit objective comparisons of performance.

To address these issues, this study recommends a unified set of evaluation metrics. The selection is guided by metric classification, the use of evaluation metrics in existing studies summarized in Table 1, and the frequency analysis of commonly used metrics shown in Figure 3b. This design ensures coverage of complementary metric categories while maintaining a balance between practical feasibility and evaluation comprehensiveness. The selected metrics and their corresponding categories are VIF (human visual quality), MI (information-based), SSIM (structural preservation), SCD (correlation-based), and SD (image feature-based). These unified metrics facilitate systematic performance comparisons across different fusion methods. Moreover, these unified metrics may provide a quantitative basis for future studies linking objective fusion quality with downstream clinical task performance, thereby supporting more clinically meaningful assessment.

## 6. Future Research Directions

### 6.1. Better Evaluation Metrics

Due to the lack of ground truth in MMIF, current evaluation methods primarily rely on subjective visual assessments and objective metrics for quantitative analysis. However, as illustrated in Table 1, different studies use inconsistent evaluation criteria, making it difficult to conduct fair and reproducible comparisons across fusion methods. This inconsistency further impedes the establishment of authoritative benchmarking systems, thereby slowing down systematic progress and methodological evolution in the field. Moreover, some metrics, such as EN, SD, and CC, typically capture only limited low-level aspects of fused images, such as contrast or statistical distribution, and often fail to reflect their overall perceptual quality [89,90].

To address these limitations, it is essential to establish unified and standardized evaluation metrics to ensure methodological fairness in comparison. Furthermore, selecting a diverse and appropriate set of metrics that are aligned with human perception and capable of capturing multiple dimensions of fusion quality is critical for the comprehensive assessment of MMIF methods.

### 6.2. Registration-Aware Robust Medical Image Fusion

Robustness to spatial misalignment is essential for the reliable clinical application of medical image fusion models. In real-world MMIF scenarios, images from different modalities are often difficult to align precisely due to differences in imaging mechanisms, acquisition protocols, and patient motion. However, most existing methods still assume well-registered inputs [17,91], while the literature on the fusion of misaligned medical images remains limited [24,92]. Future research should develop registration-aware fusion networks to improve the robustness of MMIF models to spatially inconsistent inputs.

### 6.3. Mamba-Based Architectures for Future MMIF Research

Although research on applying Mamba to MMIF is still in its early stages, the model’s ability to capture long-range dependencies with linear computational complexity positions it as a highly promising foundation for future developments. As interest in efficient sequence modeling grows, it is expected that an increasing number of Mamba-based MMIF frameworks will emerge. Notably, the design of fully Mamba-based MMIF architectures remains largely unexplored and deserves further investigation.

### 6.4. Benchmark Expansion in MMIF

The quantity and diversity of available datasets are important factors affecting MMIF model performance and generalizability in clinical applications. At present, publicly available MMIF datasets remain limited and are primarily concentrated on brain imaging and common modality combinations, such as MRI-CT, MRI-PET, and MRI-SPECT. AANLIB is the most frequently used benchmark; however, its predominant focus on brain imaging limits its applicability to broader clinical scenarios. Meanwhile, datasets covering other anatomical regions and disease types remain scarce, which constrains the development of more generalizable MMIF models.

Existing datasets also exhibit variations in image format, spatial resolution, and registration accuracy, while standardized dataset construction and evaluation protocols remain insufficiently established. Therefore, developing large-scale, standardized, and clinically diverse MMIF datasets covering multiple anatomical regions, disease types, and imaging modalities is essential for further advancing this field.

### 6.5. Application-Driven MMIF Methods

One important application of MMIF is to support downstream applications such as medical image segmentation or classification. Recently, some researchers [1] have explored deep learning-based MMIF methods designed for specific tasks. However, this direction remains relatively underexplored. We expect that task-driven MMIF methods will become a key research direction in the future.

### 6.6. Generalizable MMIF Models

Developing generalizable MMIF models remains an important future direction for adapting fusion methods to diverse clinical scenarios and fusion tasks. Early unified frameworks such as U2Fusion [15] used continual learning to handle multiple fusion tasks and alleviate subtask conflicts, but they did not promote task interaction during training. Later studies have explored different architectures to improve fusion performance and generalizability, including CNN-based methods [16,39], AE-based methods [24], GAN-based methods [18], Transformer-based methods [20,22], diffusion model-based methods [93], and Mamba-based methods [27].

However, these methods still depend on large-scale data from multiple fusion tasks and usually require task-specific retraining or multiple dedicated models, which limits their ability to achieve genuine cross-task generalization. GIFNet [57] addresses this limitation through a cross-fusion gating mechanism that introduces pixel-level supervision from digital photography fusion, promoting task-shared feature learning and enabling generalizable multi-task fusion within a single model. Future research should therefore move toward genuinely generalizable MMIF models that reduce dependence on task-specific retraining and dedicated models while enabling multiple fusion tasks within a single unified framework.

### 6.7. Clinical Applicability and Translation of MMIF

Although deep learning-based MMIF methods have shown promising performance, their clinical translation remains limited by several practical challenges. For PET/SPECT fusion, preserving quantitative information is essential because these modalities provide functional information related to radiotracer distribution, metabolic activity, and tissue uptake. Therefore, fusion methods should enhance visual quality without distorting clinically meaningful quantitative features. In addition, external validation across different institutions, scanners, imaging protocols, and patient populations remains insufficient. Practical deployment also requires compatibility with clinical infrastructure and workflows, including PACS/RIS systems, DICOM standards, radiological reading, and reporting processes. Future studies should emphasize quantitative consistency, external validation, workflow compatibility, and clinical usability to support reliable clinical translation.

## 7. Conclusions

In this review, a comprehensive overview of deep learning-based methods for MMIF is presented. It categorizes existing methods into mainstream methods and emerging methods, highlights representative models in each category, and provides a statistical analysis of loss functions, evaluation metrics, modality combinations, and dataset usage. It also reviews commonly used MMIF datasets and evaluation metrics. A significant limitation in current MMIF research lies in the inconsistency of evaluation metrics, which hampers direct and fair comparisons across methods. To address this issue, this review recommends a unified metric set to support more standardized and comprehensive assessment of MMIF methods. Moreover, this review also outlines future trends and key challenges in the field. Promising research directions include registration-aware robust medical image fusion, Mamba-based architectures, benchmark expansion in MMIF, and application-driven MMIF methods. This review serves as a useful reference for researchers in medical image fusion, enabling efficient access to prior work and guiding the design of improved MMIF methodologies.

## Figures and Tables

**Table 1:** A summary of key deep learning-based MMIF methods.

Reference	Year	Backbone	Loss Function	Modalities	General /MMIF	Metrics	Medical Dataset
CoCoNet [17]	2024	CNN	SSIM loss, Intensity similarity loss, Self-adaptive loss, Contrastive loss	MRI-PET	General	EN, SD, VIF, SF, AG, SCD	AANLIB
MM-net [32]	2024	CNN	Spatial domain loss, Frequency domain loss	MRI-SPECT	MMIF	MI, NICE, $Q_{G}$, $Q_{P}$, $Q_{C}$, $Q_{Y}$, $Q_{\mathrm{CB}}$, $Q_{\mathrm{CV}}$	AANLIB
MRSCFusion [33]	2023	CNN	Content loss, Intensity loss	MRI-CT/PET/SPECT	MMIF	SCD, MS-SSIM, $Q^{\mathrm{AB}/F}$, FMI, SF, VIF	AANLIB
SMAFusion [34]	2025	CNN	MSE loss, SSIM loss, Total variation loss	MRI-CT	MMIF	SD, SF, MI, SCD, VIF, SSIM, $Q^{\mathrm{AB}/F}$	AANLIB
FDGNet [35]	2023	CNN	Weighted fidelity loss, Feature difference loss	MRI-CT/PET/SPECT	MMIF	NMI, $Q^{\mathrm{AB}/F}$, $Q_{Y}$, VIFP	AANLIB
Fu et al. [36]	2021	CNN	MSE loss	MRI-CT/PET/SPECT	MMIF	SSIM, MI, PSNR, $Q_{\mathrm{AC}}$, TMQI, FSIM	AANLIB
MdAFuse [37]	2024	CNN	SSIM loss, Pixel loss	MRI-CT/PET/SPECT	MMIF	EN, MI, SD, VIF, SCD, SSIM, CC, MSE, rSFE	AANLIB, ADNI
MsgFusion [38]	2024	CNN	SSIM loss, Pixel loss	MRI-CT/PET/SPECT	MMIF	EN, SD, MI, RSFE, SM, VIF, RQ	AANLIB, ADNI
U2Fusion [15]	2020	CNN	SSIM loss, MSE loss, EWC loss	MRI-PET	General	SCD, CC, SSIM, PSNR	AANLIB
SDNet [16]	2021	CNN	Gradient loss, Intensity loss	MRI-PET	General	EN, FMI, MG, PSNR	AANLIB
EMFusion [4]	2021	CNN	Surface-level loss, Deep-level loss, Similarity constraints loss	MRI-CT/PET/SPECT	MMIF	SSIM, PSNR, CC, VIF	AANLIB
MUFusion [39]	2023	CNN	Content loss, Memory loss	MRI-PET	General	EN, VIF, MI, EI, $Q^{\mathrm{AB}/F}$	AANLIB
Liu et al. [40]	2021	CNN	Structure loss, Intensity loss	MRI-CT/PET/SPECT	General	VIF, AG, EN, SF, SCD, $Q^{\mathrm{AB}/F}$	Brain Atlas
Liu et al. [41]	2022	CNN	Structure loss, Intensity loss	MRI-MRI	MMIF	LMI, $Q_{Y}$, VIF, $Q^{\mathrm{AB}/F}$	AANLIB
MSAIF-net [42]	2023	CNN	Content loss, Texture loss	MRI-MRI	MMIF	CC, SSIM, $Q_{Y}$, $Q_{W}$, $Q^{\mathrm{AB}/F}$, VIF, $Q_{P}$, $Q_{\mathrm{CB}}$	BRATS, ISLES
GeSeNet [43]	2023	CNN	Edge loss, SSIM loss, Semantic loss	MRI-CT/PET/SPECT	MMIF	SSIM, SD, MI, VIF, SCD, $Q^{\mathrm{AB}/F}$	AANLIB
ECINFusion [44]	2025	CNN	Structure loss, Semantic loss, Pixel loss	MRI-CT/PET/SPECT	MMIF	NCIE, MI, $Q_{G}$, LMI, MS-SSIM, VIF, NCC, PSNR, Cosin	AANLIB
AMMNet [45]	2024	CNN	Fusion loss, Decomposition loss	MRI-CT/PET	MMIF	AG, EN, SF, MI, SD, CC, $Q^{\mathrm{AB}/F}$	AANLIB
IFSepR [46]	2023	AE	SSIM loss, MSE loss	MRI-MRI	General	EN, MG, VIF, $N_{\mathrm{abf}}$	AANLIB
UNIFusion [47]	2021	AE	SSIM loss, MSE loss, Gradient loss	MRI-PET	General	MI, SD, FMI, NCIE, $Q_{P}$, VIF	AANLIB, MS-COCO
SGFusion [48]	2023	AE	SSIM loss, Mean absolute error loss	MRI-PET	General	EN, MI, MESSIM, SCD	AANLIB, ECSSD
CDDFuse [23]	2023	AE	Gradient loss, Intensity loss, Decomposition loss, SSIM loss	MRI-CT/SPECT	General	EN, SD, SF, MIF, SCD, VIF, $Q^{\mathrm{AB}/F}$, SSIM	AANLIB
C2RF [24]	2025	AE	Fusion loss, Decomposition loss, Contrastive Learning-driven Registration loss	MRI-PET	General	MI, CC, SSIM, MSE, MEE, MAE	AANLIB
HI-net [19]	2020	GAN	Reconstruction loss	MRI-MRI	MMIF	PSNR, SSIM, NRMSE	BRATS
BMGAN [49]	2022	GAN	Adversarial loss, KL-divergence constraint, Reconstruction loss, Perceptual loss	MRI-PET	MMIF	MAE, PSNR, MS-SSIM, FID	ADNI
MGM-GAN [50]	2022	GAN	Adversarial loss, Pixel loss, Gradient loss	MRI-MRI	MMIF	PSNR, SSIM, NRMSE	BRATS
PRRGAN [51]	2024	GAN	SSIM loss, Adversarial loss, Discriminator loss	CT-MRI-SPECT, MRI- MRI-PET	MMIF	EN, SSIM, SD, PSNR, $Q_{\mathrm{CB}}$, $Q_{G}$	ADNI
DDCGAN [18]	2019	GAN	Adversarial loss, Content loss	MRI-PET	General	EN, MG, SF, SD, PSNR, CC, SSIM, VIF	AANLIB
Zhou et al. [52]	2022	GAN	Content loss, Adversarial loss, Intensity loss, Gradient loss	MRI-PET	Gneral	EN, CC, SD, SCD, $Q_{\mathrm{CB}}$	AANLIB
TCGAN [53]	2023	GAN	Content loss, Adversarial loss, SSIM loss	MRI-PET	Gneral	SSIM, PSNR, CC, SF, VIF	AANLIB
Liu et al. [1]	2022	GAN	Content loss, Adversarial loss, Discriminator loss	MRI-MRI	MMIF	MI, $Q_{G}$, $Q_{W}$, VIF	BRATS
MHW-GAN [54]	2024	GAN	Adversarial loss, Content loss, Edge detail loss, Constraint loss, Similarity loss	MRI-PET/SPECT	Gneral	$Q_{W}$, $Q_{C}$, SSIM, $N_{\mathrm{abf}}$	BRATS
MATR [21]	2022	Transformer	SSIM loss, Region-level loss	MRI-PET, GFP-PC	MMIF	MI, TE, NCIE, $Q_{G}$, $Q_{P}$, $Q_{\mathrm{CV}}$, LMI, VIF, MS-SSIM	AANLIB
MACTFusion [55]	2025	Transformer	SSIM loss, Gradient loss, Intensity loss	MRI-CT/PET/SPECT	MMIF	SCD, MS-SSIM, $Q^{\mathrm{AB}/F}$, SD, $Q_{W}$, VIF	AANLIB
FATFusion [56]	2024	Transformer	Pixel loss, Total variation loss	MRI-PET/SPECT	MMIF	MI, $Q_{G}$, $Q_{P}$, $Q_{Y}$, $Q_{\mathrm{CB}}$, VIF, $MI_{\mathrm{abf}}$	AANLIB
Cheng et al. [57]	2025	Transformer	Public loss, Private loss	MRI-PET	General	EI, VIF, SCD, AG	AANLIB
EMMA [22]	2024	Transformer	L2 loss, Sensing loss, Equivariant loss	MRI-PET	General	EN, VIF, SF, AG, SD, SCD	AANLIB
SwinFusion [20]	2022	Transformer	SSIM loss, Texture loss, Intensity loss	MRI-CT/PET	General	FMI, $Q^{\mathrm{AB}/F}$, SSIM, PSNR	AANLIB
Wang et al. [58]	2024	Transformer	MFF loss, MEF loss, Supervised loss, Unsupervised loss	MRI-CT/PET	General	EN, MI, MS-SSIM, $Q^{\mathrm{AB}/F}$, FMI, NMI, $Q_{\mathrm{CB}}$, $N_{\mathrm{abf}}$	AANLIB
DDBFusion [59]	2025	Transformer	SSIM loss, Pixel loss, Decomposition loss	MRI-PET	General	EI, SCD, CC, SF, EN	AANLIB
LFDT-fusion [60]	2025	Transformer	Diffusion loss, Fusion loss	MRI-PET/SPECT	General	VIF, EN, $Q^{\mathrm{AB}/F}$, SSIM, FMI	AANLIB
DDFM [25]	2023	Diffusion model	L1 loss	MRI-CT/PET/SPECT	General	EN, SD, MI, VIF, $Q^{\mathrm{AB}/F}$, SSIM	AANLIB
Diff-IF [26]	2024	Diffusion model	Diffusion loss, SSIM loss, Gradient loss, Intensity loss	MRI-CT	General	MI, SCD, VIF, $Q^{\mathrm{AB}/F}$, SSIM	AANLIB
FusionDiff [61]	2024	Diffusion model	Mean loss	MRI-CT	General	Qcy, EN, SF, PSNR, MI, PC	AANLIB
UUD-Fusion [62]	2024	Diffusion model	SSIM loss, Smooth absolution loss	MRI-PET	General	EI, AVG, SD, SF, VIF, EN, MI, $Q^{\mathrm{AB}/F}$	AANLIB
LPM-net [27]	2025	Mamba	SSIM loss, MSE loss, Gradient loss, Reconstruction loss	CT-MRI/PET	MMIF	SSIM, MI, SCD, VIF, $Q^{\mathrm{AB}/F}$	BRATS, IXI, RESECT, MMWHS, Prostate, HNSCC
Wu et al [63]	2025	Mamba	MSE loss, Decomposition loss, TV loss	MRI-CT/PET/SPECT	General	EN, SF, AG, SD, VIF, SCD, SSIM, MSE, $Q^{\mathrm{AB}/F}$	AANLIB
ISCDFuse [64]	2025	Mamba	SSIM loss, Texture loss, Intensity loss, Decomposition loss, MSE loss	MRI-CT/PET/SPECT	General	SSIM, VIF, SCD, MI, SF, SD, EN, AG, $Q_{W}$, MS-SSIM, $Q^{\mathrm{AB}/F}$	AANLIB
DWMFusion [65]	2025	Mamba	Wavelet loss, Contrastive loss, SSIM loss	MRI-CT/PET/SPECT	MMIF	EN, SD, MI, SF, SCD, VIF, SSIM, AG, $Q^{\mathrm{AB}/F}$	AANLIB

**Table 2:** MMIF dataset.

Dataset	Year	Organ	Modality	Disease	Download Address	Format
AANLIB	1995	Brain	CT, MRI, PET, SPECT	Normal brain, Brain tumor, Alzheimer’s disease	http://www.med.harvard.edu/AANLIB/ (accessed on 3 July 2026)	GIF
BRATS	2012	Brain	MRI	Glioma	https://www.med.upenn.edu/cbica/brats2021/ (accessed on 3 July 2026)	NIFTI
ADNI	2003	Brain	MRI, PET	Alzheimer’s disease	https://adni.loni.usc.edu/data-samples/adni-data (accessed on 3 July 2026)	-
OASIS	2010	Brain	MRI, PET	Alzheimer’s disease	https://www.oasis-brains.org/ (accessed on 3 July 2026)	NIFTI
TCIA	2014	Brain, Lungs	CT, MRI, PET, Ultrasound	Gliomas, Lung cancer	https://www.cancerimagingarchive.net/ (accessed on 3 July 2026)	DICOM

**Table 3:** Objective quantitative evaluation metrics sorted by frequency of use in the surveyed papers.

Metric	Full Name	Description	Formula	Category
VIF [78]	Visual information fidelity	It measures the preservation of visual information in the fused image.	$VIF\left(I_{V},I_{R},I_{F}\right)=\sum_{k}p_{k}\frac{\sum_{b}FVID_{s,b}\left(I_{V},I_{R},I_{F}\right)}{\sum_{b}FVIND_{s,b}\left(I_{V},I_{R},I_{F}\right)}$	Human visual quality metrics
MI [79]	Mutual information	It measures the degree of information shared between the fused image and its source images.	$\begin{array}{cc} MI & =MI_{A,F}+MI_{B,F}\\ MI_{X,F} & =\sum_{x,f}p_{X,F}\left(x,f\right)\log\frac{p_{X,F}\left(x,f\right)}{p_{X}\left(x\right)p_{F}\left(f\right)} \end{array}$	Information-based metrics
SSIM [80]	Structural similarity index measure	It measures the similarity between the source and fused images by comparing their structural information.	$\begin{array}{cc} SSIM & =SSIM_{A,F}+SSIM_{B,F}\\ SSIM_{X,F} & =\frac{2\mu_{x}\mu_{f}+C_{1}}{\mu_{x}^{2}+\mu_{f}^{2}+C_{1}}\cdot \frac{2\sigma_{x}\sigma_{f}+C_{2}}{\sigma_{x}^{2}+\sigma_{f}^{2}+C_{2}}\cdot \frac{\sigma_{xf}+C_{3}}{\sigma_{x}\sigma_{f}+C_{3}} \end{array}$	Structural preservation metrics
EN [81]	Entropy	It measures the information content of the fused image, reflecting the complexity and diversity of its pixel distribution.	$EN=-\sum_{l=0}^{L-1}p_{l}\log_{2}p_{l}$	Information-based metrics
$Q^{\mathrm{AB}/F}$ [82]	Gradient-based fusion performance	It measures how much edge information from the source images is retained in the fused image.	$Q^{AB/F}=\frac{\sum_{i=1}^{N}\sum_{j=1}^{M}\left(Q^{AF}\left(i,j\right)w^{A}\left(i,j\right)+Q^{BF}\left(i,j\right)w^{B}\left(i,j\right)\right)}{\sum_{i=1}^{N}\sum_{j=1}^{M}\left(w^{A}\left(i,j\right)+w^{B}\left(i,j\right)\right)}$	Information-based metrics
SCD	Sum of the correlations of differences	It evaluates the quality of a fused image by measuring the correlation between each source image and its corresponding difference image with the fused result.	$\begin{array}{cc} SCD & =r\left(I_{V},D_{V,F}\right)+r\left(I_{R},D_{R,F}\right) \end{array}$	Correlation-based metrics
SD [83]	Standard deviation	The standard deviation metric measures the distribution and contrast of the fused image.	$SD=\sqrt{\sum_{i=1}^{M}\sum_{j=1}^{N}{\left(F\left(i,j\right)-\mu \right)}^{2}}$	Image Feature-based metrics
SF [84]	Spatial frequency	It is an index that indicates the variation in grayscale values within an image. It reflects the level of detail and texture present.	$\begin{array}{cc} \mathrm{SF} & =\sqrt{H^{2}+V^{2}}\\ H & =\sqrt{\frac{1}{MN}\sum_{i=1}^{M}\sum_{j=2}^{N}{\left|I_{F}\left(i,j\right)-I_{F}\left(i,j-1\right)\right|}^{2}}\\ V & =\sqrt{\frac{1}{MN}\sum_{i=2}^{M}\sum_{j=1}^{N}{\left|I_{F}\left(i,j\right)-I_{F}\left(i-1,j\right)\right|}^{2}} \end{array}$	Image feature-based metrics
PSNR [85]	Peak signal-to-noise ratio	It reflects the level of distortion in the fused image by calculating the ratio between peak signal power and noise power.	$PSNR=10\log_{10}\frac{r^{2}}{MSE}$	Information-based metrics
CC [86]	Correlation coefficient	It evaluates the strength of the linear relationship between the fused result and the original inputs.	$\begin{array}{cc} CC & =\frac{r_{AF}+r_{BF}}{2} \end{array}$	Correlation-based metrics

**Table 4:** Quantitative comparison results of different MMIF methods.

Method	VIF	MI	SSIM	SD	SCD	EN	$Q^{\mathrm{AB}/F}$	SF	PSNR	CC
DDCGAN	0.461	1.788	0.120	48.230	0.492	5.454	0.275	18.110	12.230	0.786
U2Fusion	0.468	1.796	0.542	59.466	0.531	5.303	0.402	20.961	14.993	0.860
EMFusion	0.655	2.113	1.270	80.298	0.893	5.271	0.714	33.365	13.602	0.798
C2RF	0.730	2.307	1.266	90.569	1.409	5.298	0.729	36.714	12.576	0.798
CDDFuse	0.715	2.337	1.287	93.250	1.591	5.287	0.724	37.953	12.415	0.806
EMMA	0.516	1.776	0.683	90.375	1.502	5.979	0.571	28.261	12.957	0.833
SwinFusion	0.747	2.234	0.657	88.406	1.384	5.782	0.753	37.135	12.212	0.806
Diff-IF	0.772	2.611	1.297	92.001	1.438	5.055	0.716	37.356	12.528	0.806
DDFM	0.643	2.611	1.128	75.346	1.234	5.043	0.509	22.492	15.267	0.869

## Data Availability

No new original data were generated.

## Abbreviations

The following abbreviations are used in this manuscript:

MMIF	Multimodal medical image fusion
MRI	Magnetic resonance imaging
CT	Computed tomography
PET	Positron emission tomography
SPECT	Single-photon emission computed tomography
DL	Deep learning
CNN	Convolutional neural network
AE	Autoencoder
GAN	Generative adversarial network
INN	Invertible neural network
SSM	Structured state space model
DICOM	Digital Imaging and Communications in Medicine
PACS	Picture archiving and communication system
RIS	Radiology information system
VIF	Visual information fidelity
MI	Mutual information
SSIM	Structural similarity index measure
SCD	Sum of correlations of differences
SD	Standard deviation
EN	Entropy
SF	Spatial frequency
AG	Average gradient
CC	Correlation coefficient
PSNR	Peak signal-to-noise ratio
MSE	Mean squared error
